# Pre-Clinical Pregnancy Models for Evaluating Zika Vaccines

**DOI:** 10.3390/tropicalmed4020058

**Published:** 2019-04-07

**Authors:** In-Jeong Kim, Marcia A. Blackman, Jr-Shiuan Lin

**Affiliations:** Trudeau Institute, Saranac Lake, NY 12983, USA; ijkim@trudeauinstitute.org (I.-J.K.); mblackman@trudeauinstitute.org (M.A.B.)

**Keywords:** Zika virus, ZIKV, congenital Zika syndrome, vaccine, pre-clinical pregnancy model, mouse, non-human primate

## Abstract

Zika virus (ZIKV) infection during pregnancy can result in a variety of developmental abnormalities in the fetus, referred to as Congenital Zika Syndrome (CZS). The effects of CZS can range from the loss of the viable fetus to a variety of neurological defects in full-term infants, including microcephaly. The clinical importance of ZIKV-induced CZS has driven an intense effort to develop effective vaccines. Consequently, there are approximately 45 different ZIKV vaccine candidates at various stages of development with several undergoing phase I and II clinical trials. These vaccine candidates have been shown to effectively prevent infection in adult animal models, however, there has been less extensive testing for their ability to block vertical transmission to the fetus during pregnancy or prevent the development of CZS. In addition, it is becoming increasingly difficult to test vaccines in the field as the intensity of the ZIKV epidemic has declined precipitously, making clinical endpoint studies difficult. These ethical and practical challenges in determining efficacy of ZIKV vaccine candidates in preventing CZS have led to increased emphasis on pre-clinical testing in animal pregnancy models. Here we review the current status of pre-clinical pregnancy models for testing the ability of ZIKV vaccines to prevent CZS.

## 1. Introduction

Zika virus (ZIKV) is a mosquito-borne flavivirus that was first identified in the Zika forest of Uganda in 1947 [1]. Outbreaks of Zika occurred in 2007 in the Micronesia island of Yap [2] and 2013 in French Polynesia [3], but caused little concern, as they were largely asymptomatic with only occasional mild-febrile symptoms including fever, rash, myalgia, and conjunctivitis. In rare cases, ZIKV infection was also associated with the development of neuroimmunological disorders such as Guillain–Barre syndrome in adults [4,5,6]. However, the true clinical significance of ZIKV was first appreciated during the 2015 outbreak in Brazil, which attracted world-wide attention due to its association with increased frequencies of babies born with microcephaly [7,8]. ZIKV hence becomes the newest TORCH infections, which include toxoplasmosis, others (including syphilis, listeriosis, varicella, and parvovirus B19), rubella, cytomegalovirus, and herpes simplex virus [9]. 

## 2. Consequences of ZIKV during Pregnancy for the Developing Fetus

The pathology induced by ZIKV infection during pregnancy is termed congenital Zika syndrome, or CZS. CZS is associated with a vast spectrum of consequences, ranging from loss of the viable fetus to a variety of neurological defects in full-term infants, including microcephaly, craniofacial disproportion, spasticity, seizures, and ocular and hearing abnormalities [10,11]. It has also been noted that some infants born from ZIKV-infected mothers can appear neurologically normal at birth, but manifest developmental issues over time [12]. The devastating developmental abnormalities in infants born to women infected with ZIKV during pregnancy prompted the World Health Organization to declare Zika a Public Health Emergency of International Concern in 2016 [13]. This designation spurred an intense effort to develop ZIKV vaccines [4,14]. 

## 3. Target Product Profile (TPP) for Vaccine Development

The WHO Target Product Profile (TPP) for ZIKV vaccines identifies the preferred target for vaccination as women of childbearing age, potentially including pregnant women [15,16]. The goal is to protect the fetus from infection during pregnancy, and to prevent, or minimize, the congenital abnormalities associated with CZS. Thus, it is critically important to demonstrate that vaccines not only prevent infection of adults, but that the protection induced by vaccination prior to pregnancy will prevent CZS during ensuing pregnancy. If pregnant women become primary targets for vaccination, it will be particularly challenging to fulfill safety and efficacy requirements for field trials [17]. An additional challenge will be to induce durable immunity in women prior to pregnancy, so that it remains effective during later pregnancy. Detection of virus in semen samples collected from men more than two months after infection points to a reservoir of ZIKV that can be sexually transmitted [18,19,20,21,22,23,24,25]. Thus, men are a secondary target population in the context of an emergency outbreak because of their ability to transmit virus. 

Many reviews have focused on the development of ZIKV vaccines exclusively in terms of their ability to block ZIKV infection [17,26,27,28,29,30,31]. Here, we will focus on pre-clinical pregnancy models to evaluate candidate vaccines for their ability to prevent vertical transfer of virus to the fetus during pregnancy and the devastating manifestations of CZS. 

## 4. Challenges for Clinical Testing of Anti-ZIKV Vaccines

Currently over 45 ZIKV vaccine candidates are under development (Table 1). They have been tested in animal models and have been shown to be efficacious in preventing ZIKV infection of adult animals. While several of these candidates have advanced to clinical trials, it has proven challenging to clinically evaluate them for their ability to prevent CZS. First, the intensity of the ZIKV epidemic has declined dramatically, from a peak weekly new infection rate of >25,000 in 2016 to <1000 in May, 2017 [32,33,34,35,36]. This not only lessens the urgency for vaccine development, but also makes clinical endpoint studies difficult, as vaccination cohorts need to be substantially larger. Furthermore, Zika is now endemic in many areas, making it difficult to identify adequate numbers of ZIKV-naïve individuals. In addition, the low incidence of symptoms during acute disease and the low frequency of sequelae such as Guillain–Barre syndrome makes endpoints difficult to determine in clinical trials [34,37].

Second, other flaviviruses, such as dengue virus (DENV) and West Nile virus (WNV), co-exist with ZIKV in the same geographical regions, raising the possibility that cross-reactive immunity among flaviviruses may interfere with ZIKV vaccine efficacy. Indeed, several studies support this possibility [64,65]. For example, *in vitro* studies have suggested that pre-existing DENV-specific antibodies are capable of mediating antibody-dependent enhancement (ADE) of ZIKV infection [64]. Although ADE has been difficult to confirm *in vivo*, a recent study in pregnant mice showed that DENV-specific antibodies increased placental damage, growth restriction, and fetal resorption during a ZIKV infection in mice that are deficient in signal transducer and activator of transcription (STAT) 2 gene [65]. In addition, enhanced replication of ZIKV was reported in human placental tissue in the presence of DENV-specific antibodies [65]. Thus, ZIKV-vaccination trials in DENV-endemic areas must consider the possibility that pre-existing DENV-specific antibodies may complicate vaccination outcomes, similar to the safety issues that recently occurred in the trial of Dengvaxia, a DENV vaccine [66,67]. The potential risk of ADE means that extensive pre-screening will be required to determine DENV- and ZIKV- immune status in ZIKV human vaccination trials. 

Third, although the TPP includes the possibility of testing ZIKV vaccine candidates in pregnant women, this subgroup has historically been excluded from clinical trials for safety and ethical reasons [30,68,69,70,71]. There has been some discussion to allow pregnant women to participate in clinical trials. But even if such trials are ultimately permitted, it is likely that the challenges will be extensive. Difficulties associated with developing an adequate model for informed consent and completing enrollment quotas will deter developers from sponsoring trials. 

One possible solution to these various challenges to clinical trials would be to develop human challenge models using volunteers. However, human ZIKV infection studies were recently placed on a temporary hold due in part to our incomplete understanding of persistent reservoirs of ZIKV [18]. One reason for concern was that ZIKV and ZIKV RNA can persist in semen for several months in both symptomatic and asymptomatic men, can be harbored in the testes and prostate gland, and can be sexually transmitted [18,19,20,21,22,23,24,25]. This hold has now been partially reversed and a human infection trial has been approved by the NIH to test safety and immunogenicity of a live attenuated Zika vaccine, rZIKV/D4Δ30-713, in 28 flavivirus-naïve individuals (clinical trial NCT03611946). Nevertheless, it is highly unlikely that human infection models will be approved to test vaccine efficacy in pregnant women.

A less problematic solution is to test vaccines in pre-clinical animal pregnancy models, which is the focus of this review. Here, we discuss the currently available pre-clinical animal pregnancy models used for testing vaccine efficacy to prevent CZS and the vaccines that have been tested in these models. Although there are limitations to the direct relevance of pre-clinical studies for humans, safety and efficacy studies in animals are considered essential for reducing risk in clinical studies [72]. Much information can be obtained from animal models to allow better-informed clinical studies with reduced risk. 

## 5. Pregnant Animal Models of ZIKV Infection

### 5.1. Pregnant Mouse Models

Ever since the 2015 ZIKV outbreak in Brazil which was associated with microcephaly, numerous efforts have been devoted to developing animal models of ZIKV infection that can recapitulate the clinical course of human infection, especially during pregnancy. Animal models of ZIKV infection during pregnancy has been comprehensively reviewed in recent publications [73,74,75,76]. There are advantages, disadvantages, and limitations of various animal models. Mouse is the most widely used species in human disease models due to its low cost, ease of breeding, handling, and manipulation, and the availability of numerous tools, reagents, and resources. However, in the context of pregnancy, the mouse has the disadvantage of a relatively short gestation period and a fundamentally different placental architecture than NHP and human [77,78,79,80]. Mouse, NHP and human all possess a hemochorial placenta in which the maternal blood is in direct contact with the chorion. However, the mouse placenta is hemotrichorial, with three trophoblast layers that separates the fetal blood supply from the maternal blood supply, and matures relatively late in gestation. In contrast, NHP and human have a hemomonochorial placental architecture, with a single layer of syncytiotrophoblast which develops early in pregnancy [77,78,79,80]. Antibody transport across the placenta by neonatal Fc receptor for IgG (FcRn) is also different between mouse and NHP/human [81]. These differences limit the translation of findings in the mouse model to the clinic. In addition, the mouse is less susceptible to ZIKV infection than NHP and human because ZIKV is unable to bind to mouse Stat2 and thus cannot block the mouse innate type I interferon (IFN) response [82]. Therefore, early mouse models of ZIKV infection employed immunocompromised mice genetically lacking Type I IFN signaling or wild-type mice treated with antibodies to block Type I IFN signals [83,84,85,86]. Alternatively, viruses were inoculated via non-physiological routes to bypass the maternal–fetal interface [87,88,89,90,91,92], or delivered in very high dose systemically [93]. These studies successfully established maternal infection, demonstrated vertical transmission and placental pathology, and reproduced adverse fetal outcome including fetal demise, intrauterine growth restriction (IUGR) and neurological damage. However, they did not allow us to fully understand the role of the immune response to ZIKV infection during pregnancy, nor vertical transmission in a more physiologically relevant setting. 

The critical role of STAT2 in ZIKV pathogenesis, specifically in pregnancy, has been demonstrated in two models: human STAT2 knock-in (hSTAT2-KI) mice [94] and Stat2 knockout (*Stat2−/−*) mice [65]. In human STAT2 knock-in mice, mouse *Stat2* gene was replaced by the human *STAT2* gene but the mouse *Stat2* promoter was retained. Subcutaneous inoculation of pregnant hSTAT2-KI mice with a mouse-adapted virus ZIKV-Dak-MA (which appears to be more virulent than the parental strain) resulted in significantly higher levels of ZIKV RNA in maternal serum, spleen, placenta, and fetal head than pregnant wild-type mice at 7 days post infection (dpi). This otherwise immunocompetent mouse model demonstrated vertical transmission and fetal infection. Similarly, intramuscular inoculation of pregnant *Stat2^−/−^* mice with ZIKV strain PRVABC59 resulted in ZIKV RNA presence in maternal blood, brain and spinal cord at 3 dpi, and fetal growth restriction and fetal demise at 7 dpi. 

While immunocompromised mouse models have been useful in recapitulating vertical transmission and the neurotropic nature of ZIKV infection and in demonstrating a critical role of Type I and Type III IFN signaling in ZIKV pathogenesis, they have limitations for studying the impacts of maternal and fetal immune response to ZIKV pathogenesis and pregnancy outcome. Indeed, studies in immunocompetent mouse models revealed a potential detrimental role of the immune response to ZIKV during pregnancy [95]. Using a specific breeding scheme (*Ifnar1^−/−^* dams mated to *Ifnar^+/−^* sires) and intravaginal infection, Yockey *et al.* showed that fetuses that expressed IFNAR (*Ifnar1^+/−^*) were resorbed after ZIKV infection during early pregnancy despite relatively lower viral titer in their placentas, whereas their *Ifnar1^−/−^* littermates that did not express IFNAR continued to develop. This implied that while Type I IFN signaling is considered to be protective in ZIKV infection, it could mediate abnormal placental development and adverse fetal outcome if the fetuses have the ability to respond to Type I IFN. 

Studies in pregnant immunocompetent mouse models also revealed the vulnerability of the fetus during maternal ZIKV infection [96]. While ZIKV RNA was detected in maternal serum at 1-2 dpi and in the spleen for up to 8 dpi after intravenous inoculation, ZIKA RNA was rarely detected in placentas and fetuses. However, even without apparent placental and fetal infection, maternal exposure to ZIKV nonetheless caused placental pathology and profound fetal abnormalities, including high frequency of fetal demise. Maternal or fetal viral load did not correlate with adverse fetal outcome. Instead, placental insufficiency and pathology appeared to contribute to fetal abnormalities [95,96]. The observation that adverse fetal outcome may occur even in the absence of vertical transmission raise the possibility that ZIKV countermeasures that can effectively block vertical transmission of the virus still may not effectively prevent CZS. 

Overall, despite the abovementioned disadvantages and limitations, existing pregnant mouse models of ZIKV infection have recapitulated different aspects of several disease manifestations observed in humans, including fetal demise, IUGR, placental and fetal pathology, and have provided insight into the mechanisms of ZIKV pathogenesis and fetal outcome. Early studies on vaccine efficacy using pregnant mouse models have been encouraging. Thus, mouse models of ZIKV infection during pregnancy are highly valuable in evaluating vaccine efficacy and therapeutics during early developmental pre-clinical phases. 

### 5.2. Pregnant Non-Human Primate (NHP) Models

In contrast to rodents, the non-human primates (NHPs) are susceptible to ZIKV infection and share greater similarity in placental architecture and pregnancy with human. Thus, ZIKV infection in pregnant NHP is a well-suited translational model and has recapitulated the complex pathogenesis of ZIKV infection including vertical transmission, intrauterine fetal death, fetal neuropathology, and placental pathology. Rhesus macaque (*Macacca mulatta*) [97,98,99,100,101,102,103,104], pigtail macaque (*Macacca nemestrina*) [105,106], common marmoset (*Callithrix jacchus*) [107], and more recently, olive baboons (*Papio anubis*) [108], have been used to study ZIKV pathogenesis and teratogenicity in pregnancy. These studies consistently observed prolonged viremia in pregnant animals (up to 70 dpi) compared to non-pregnant animals, which typically resolved in 7-10 days. While viremia in pregnant macaques usually developed 1–2 days post infection, the onset of viremia in pregnant baboons was slightly delayed and peaked at day 7 [108]. Despite the persistent viremia, most ZIKV-infected pregnant NHPs did not exhibit substantial clinical signs, which closely resembles asymptomatic or mildly symptomatic human ZIKV infections. This raises the concern that ZIKV-associated fetal adverse outcome may occur regardless of maternal clinical symptoms, and therefore may be under-reported in humans because a majority of ZIKV infections (~60–80%) are asymptomatic. A recent aggregated analysis of ZIKV-infected pregnant NHPs at six National Primate Research Centers indicated that fetal death occurs in 26% of ZIKV-infected pregnant macaques [103], in comparison to 5.8% miscarriage rate and 1.6% still birth rate reported in symptomatic women [109]. Importantly, along with mouse models, these studies reveal that maternal clinical symptoms are not a reliable indicator of vaccine efficacy. 

Vertical transmission of ZIKV in pregnant NHP appears to be very effective: nearly 100% in rhesus macaque and marmoset, and 75% in baboons, with a diverse range of fetal pathology. The most devastating outcome, fetal loss, has been observed in rhesus macaque (13 of 50 animals) [100,101,102,103], marmoset (2 of 2 animals) [107], and baboon (1 of 4 animals) [108]. ZIKV RNA was detected in placenta, amniotic fluid and various fetal tissues. ZIKV-infected pregnant rhesus macaques and marmosets exhibited not only high levels of ZIKV RNA as well as infectious virus in the placentas, but also severe placental pathology [99,101,107]. These studies suggest that placental damage and insufficiency is associated with fetal infection and may contribute to the development of fetal neuropathology. A pregnant rhesus macaque model which combined maternal intravenous and fetal intra-amniotic ZIKV inoculations indicated that ZIKV RNA showed different tissue tropism in dams and fetuses [102]. While ZIKV RNA was detected in most lymphoid tissues of the dams, ZIKV RNA was predominantly detected in neural tissues of the fetus and neonate. Although gross microcephaly was never observed in NHP, congenital ZIKV infection of rhesus macaque, pigtail macaque, and baboon caused fetal neuropathology that reproduces fetal neurological diseases observed in human CZS [101,102,105,106,108]. Fetal neuropathology includes brain lesions, brain calcification, and apoptosis of neural progenitor cells. In pregnant pigtail macaques, ZIKV infection can cause a spectrum of subtle fetal brain injuries in the absence of microcephaly, raising the concerns that standard prenatal diagnosis may not be able to detect “silent pathology” in the fetal brain, and that there may be long-term neurological defects in neonates that appear to be normal at birth [106]. 

Robust immune responses were also observed in ZIKV-infected pregnant rhesus macaques, marmosets and baboons [99,104,107,108]. Maternal type I/II interferon-associated genes and proinflammatory cytokines were demonstrated as early as day 2 post inoculation in marmosets [107], which is earlier than male marmosets [110]. A robust proinflammatory fetal immune response was also observed in *in utero* ZIKV infection of rhesus macaque [99]. ZIKV-specific maternal antibodies were detected as early as 6 dpi, lasted for 2-3 weeks and exhibited neutralizing activity for up to 12 weeks [99,108]. A recent publication used high-density peptide microarrays to profile anti-ZIKV antibody reactivity in pregnant and non-pregnant rhesus macaque [104]. It confirmed that a ZIKV-specific IgG antibody response targeted a conserved epitope of ZIKV nonstructural protein 2B (NS2B). The antibody reactivity of NS2B_1427-1451_RD25 in pregnant animals can be detected as late as 18 weeks post infection, and shares cross-reactivity with DENV. Despite the early systemic and robust proinflammatory and antibody responses, pregnant animals nevertheless experienced prolonged viremia and placental and fetal infection. This immune response and the observed pathology likely reflect the enhanced susceptibility of pregnant versus non-pregnant individuals in ZIKV infection, and the inefficient protection or even pathological effects of immune responses. Further studies are warranted to determine the exact roles of maternal–placental–fetal immune responses in ZIKV infection during pregnancy in both mouse and NHP models. 

## 6. Protective ZIKV Vaccines Against CZS During Pregnancy Using Mouse Models 

To date, many candidate ZIKV vaccines have been assessed for their capacity to prevent infection (Table 1). However, since the major goal of ZIKV vaccines is to protect the fetus during pregnancy, candidate vaccines must also be assessed for their ability to block vertical transmission of virus and prevent the development of CZS during pregnancy. As previously discussed, it is not possible to test the vaccines in clinical trials or human infection models involving pregnant women. Therefore, pre-clinical animal pregnancy models are beginning to play an essential role in ZIKV vaccine development.

Multiple vaccine platforms, including live attenuated vaccines (LAV), chimeric virus vaccines, and RNA vaccines have been tested in pre-clinical mouse pregnancy models (Table 2). In general, most of these vaccines were effective at reducing or preventing vertical transmission of virus in mice, in terms of reduced infection of the placenta, fetus and/or fetal brain. However, to date, none of these vaccine candidates have been tested in NHP pregnancy models, which are more reflective of the human situation. Thus, the challenge of the future is to test promising vaccine candidates in the more technically demanding NHP pregnancy models. In addition, it will be important to determine vaccine efficacy at protecting the fetus when the vaccine is delivered prior to pregnancy and how long such protection persists. 

## 7. Goals for Vaccination to Prevent CZS

The Zika TPP indicates that the vaccine target should be women of child-bearing age, perhaps pregnant women, and, if possible, men (to eliminate the possibility of sexual transmission). Current vaccination and antibody transfer studies suggest that the correlate of protection for ZIKV infection is neutralizing antibody. However, vaccine efficacy must also be assessed in terms of preventing CZS during pregnancy; in this case, the correlates of protection may include cellular as well as humoral immunity. Some important questions that can be addressed in pre-clinical pregnancy models are:

1. Will prophylactic vaccination of the mother elicit maternal immunity that can prevent ZIKV-induced fetal demise throughout pregnancy?
2. Is antibody the correlate of protection against fetal demise? Is pre-existing antibody prior to pregnancy sufficient for protection of the developing fetus? What are the quantitative and qualitative characteristics of antibody necessary for protection?
3. Will transfer of antibodies from vaccinated or infected humans protect against fetal demise?
4. Is elimination of peripheral viremia or viral RNA an adequate determinant of fetal protection?
5. Is prevention of vertical transmission sufficient for protection against fetal demise? Or can virus-induced placental damage result in fetal demise?
6. Can pre-existing DENV antibodies enhance transport of ZIKV across the placenta by a mechanism of ADE?

These questions can be adequately addressed for current vaccines using pre-clinical pregnancy models, which are currently under development. Pre-clinical experiments can determine theoretical risk of clinical studies in pregnant women. A logical progression is studies in pre-clinical pregnancy models to well-controlled human pregnancy challenge models to vaccination of pregnant women in the clinic. 

## Figures and Tables

**Table 1:** Zika virus (ZIKV) vaccine candidates and platforms.

Vaccine Platform	Status	Developer/Sponsor	Backbone ^1^ (Licensed)	Advantage	Disadvantage	Ref.
**Live attenuated**						
Pentavalent DENV/ZIKV	Unknown	Takeda	N/A	Established commercial platforms available; Establish potent and enduring immunity; rapid generation and manufacturing; gain stability with advanced gene manipulation techniques	Not recommended for pregnant women and pediatric applications in young children for safety reasons	N/A
Truncated ZIKV E90	Pre-clinical	BIME, China	JEV			[38]
ChinZIKV	Pre-clinical	BIME, China	JEV			[39]
rZIKV/D4Δ30-713	Ph1, NCT03611946	NIAID/NIH	N/A			N/A
ZIKV-3′UTR-Δ10 & ZIKV-3′UTR-Δ20	Pre-clinical	UTMB/PAHO	N/A			[40,41]
ZIKV-NS1-DKO	Pre-clinical	UTMB	N/A			[42]
ZIKV-C7a/t-LAV	Pre-clinical	UTMB	N/A			[43]
Codon pair-deoptimized ZIKV	Pre-clinical	Chinese Academy of Science, China	N/A			[44]
**Purified inactivated**						
ZPIV	Ph1, NCT02963909; NCT02952833; NCT02937233; NCT03008122	WRAIR/BIDMC	N/A	Existing commercial platforms; safe, easy to formulate in combination with adjuvants; elicit robust antibody response	Require high concentration of purified virus; inactivation may lose conformational epitopes; weak T cell-mediated immunity	[45,46,47]
PIZV (TAK-426)	Ph1, NCT03343626	Takeda	N/A			N/A
VLA1601	Ph1, NCT03425149	Valenva Austria GmbH/Emergent BioSolution	N/A			N/A
**Viral Vector**						
Ad26-ZIKV.001	Ph1, NCT03356561	Janssen Vaccine and Prevention B.V.	N/A	Relatively stable, easy acquisition of high titer virus; Scalable manufacturing, safe, strong immunogenicity, self-adjuvanticity	Safety concerns in pregnant women; Risks of possible revertant generation; Possible complications with pre-existing immunity	[48]
RhAd52-ZIKVPrM/E	Pre-Clinical	BIDMC	N/A			[45,46]
AdC7-M/E	Pre-Clinical	Univ. of Chinese Academy of Sciences, China	N/A			[49]
Ad5-Sig-prM-Env Ad5-Env	Pre-Clinical	Beijing Institute of Biotechnology, China	N/A			[50]
MMRV/CHIKV and ZIKV	Pre-clinical	Yale University	MMRV			[51]
rVSV-ZIKVprM/E and VSV-ZIKVprMsolE	Pre-clinical	NIH	N/A			[52]
VSV-prM-E-NS1	Pre-clinical	Ohio State Univ	N/A			[53]
MV-ZIKV	Ph1, NCT02996890	Themis Bioscience GmbH	MV			N/A
ChimeriVax-Zika (CYZ)	Pre-clinical	Sanofi Pasteur	YFV-17D			[54]
YF-ZIKprM/E	Pre-clinical	Rega Institute, Belgium	YFV-17D			[55]
**Nucleic Acids: DNA or RNA**						
pDNA: GL5700	Ph1, NCT02809443; NCT02887482	Inovio/GeneOne	N/A	Rapid manufacturing platform—‘plug and play’ Safe: incapability of integrating in the host genome mRNA by itself is not-immunogenic, not infectious	No DNA or RNA vaccines licensed for human use. Delivery of vaccine (e.g., electroporation) may increase cost; Require stable, effective delivery platforms; Degradation by ribonuclease	[56,57]
VRC-ZKADNA085-00-VP (VRC5288)	Ph1, NCT02840487	NIAID/VRC	N/A			[58,59]
VRC-ZKADNA090-00-VP (VRC-5283)	Ph1, NCT02996461; Ph2, NCT03110770	NIAID/VRC	N/A			[58,59]
VLP CprME/NS2B/NS3	Pre-clinical	Technovax	N/A			[60]
mRNA 1325	Ph1/2, NCT03014089	Moderna Therapeutics	N/A			[42,61]
mRNA-LNP	Pre-clinical	University of Pennsylvania	N/A			[62]
pZIKV-3’UTR-Δ20	Pre-clinical	UTMB	N/A			[63]

^1^ A constituent of ZIKV vaccine is based on live-attenuated vaccine that has been licensed previously. N/A, not applicable. Abbreviation: Ad, Adenovirus; BIDMC, Beth Israel Deaconess Medical Center; BIME, Beijing Institute of Microbiology and Epidemiology; C, Capsid; CHIKV, Chikungunya Virus, DNA, deoxynucleic acid; DENV, Dengue Virus; sE, soluble Envelope protein; JEV, Japanese Encephalitis Virus; LNP, Lipid Nanoparticle; MMRV, Measles, Mumps, Rubella Vaccine; mRNA, messenger ribonucleic acid; MV, Measles Virus; NIH, National Institutes of Health; NS1, Non-Structural protein 1; NS2B, Non-Structural protein 2B; NS3, Non-Structural protein 3; prM/E, proprotein of Membrane and Envelope; PATO, Pan American Health Organization; PIZV, Purified Inactivated Zika Virus; Sig, Signaling sequence; UTMB, University of Texas Medical Branch; VLP, virus-like particle; VRC, Vaccine Research Center; VSV, Vesicular Stomatitis Virus; WRAIR, Walter-Reed Army Institute of Research; YFV, Yellow Fever Virus; ZIKV, Zika Virus; ZPIV, Zika Purified Inactivated Virus.

**Table 2:** Vaccines protective against ZIKV infection during mouse pregnancy.

Vaccine	Target ZIKV Protein	Dam × Sire	Vaccination Dose	Challenge Dose	Challenge Strain	Outcomes	Ref.
**Live attenuated vaccine**							
ZIKV-NS1-DKO	Deletion of 2 glycosylation sites in NS1	C57BL/6 × C57BL/6	10^5^ PFU, s.c. ^1^	10^5^ PFU, s.c. at E6 ^3^	Mouse-adapted Dakar 41519	Reduced viral loads in placenta and fetal heads; reduced placental damage and fetal demise	[42]
ZIKV-3′UTR-D10	Deletion of 10 aa at 3′UTR	C57BL/6 × C57BL/6	10^5^ FFU, s.c. ^1^	10^5^ FFU, s.c. at E6 ^3^	Mouse-adapted Dakar 41519	Reduced viral loads in the placentas and fetal heads	[40,41]
ZIKV-C7a/t-LAV	Deletion of 9 aa in C	A129 × A129	10^5^ FFU, s.c.	10^6^ PFU, s.c. at E10.5	PRVABC59	No detectable viremia in dams; prevent vertical transmission to fetuses; VNAb was detected in fetal blood	[43]
Codon pair-deoptimized ZIKV	Codon pair-deoptimization in E and NS1	AG6 × AG6	10^2^ IFU, i.p.	10^4^ IFU, i.p. at E6	Asian-linage strain SZ-WIV01	Protected from fetal demise; viral loads in fetuses were not determined	[44]
ChinZIKV	prM/E	Balb/c × Balb/c	10^4^ PFU, s.c.	10^5^ PFU, i.p. or s.c. at E6 ^3^ or E13.5 ^3^	Clinical isolate GZ01 strain ^4^	Induced durable immunity; protected dams and fetuses from virus replication, vertical transmission and fetal demise; All neonates born from the vaccinated dams survived after lethal challenge	[39]
**Chimeric virus vaccine**							
YF-ZIKprM/E	Capsid anchor -prM/E	NMRI × NMRI	10^4^ PFU, i.p. ^2^	1 × 10^5^ TCID_50_, IPL at E12.5	French Polynesian strain H/PF13	No detectable virus in dams and pups; no brain pathology after challenge.	[55]
MV-ZIKV-sE	prM/E-sE (no TM domain)	*Ifnar^−/−^* -CD46Ge × Balb/C	5 × 10^4^ TCID_50_, i.p.	10^3^ TCID_50_, s.c. at E7.5	PF/2013/251013-18	Reduced viral loads in the placenta; no detectable viral RNA in fetal brains; no fetal demise	[111]
**DNA/RNA vaccine**							
mRNA-LNP	PrM/E	C57BL/6 × C57BL/6	2 × 10 ug, i.m. ^2^	10^5^ FFU, s.c at E6 ^3^	mouse-adapted Dakar 41519	Reduced viral loads in dams, placentas, and fetal heads	[42]
pZIKV-3’UTR-Δ20	Deletion of 20 aa at 3′UTR	A129 × A129	1 ug, i.m.	10^6^ FFU, s.c at E10.5	PRVABC59	No detectable viral RNA in the placentas and fetal heads	[63]

^1^ Pre-vaccine treatment of mice with 0.5 mg anti-Ifnar1 Ab, i.p. administration at one day prior to vaccination. ^2^ Pre-vaccine treatment of mice with 2 mg anti-Ifnar1 Ab, i.p. administration at one day prior to vaccination. ^3^ Pre-challenge treatment of mice with 2 mg anti-Ifnar1 Ab, i.p. administration at one day prior to virus challenge. ^4^ Gene Bank Accession Number, KU820898. Abbreviations: AG6, Interferon-α/β/γ triple knock-out mouse strain; C, Capsid protein; sE, soluble Envelope protein; Ifnar1 Ab, Type 1interferon receptor antibody; IFU, Inclusion-Forming Unit; i.m, intramuscular; IPL: intra-placenta; JEV, Japanese Encephalitis Virus; LNP, lipid nanoparticle, MV, Measles virus; NS1, Non-Structural protein 1; prM/E, proprotein of Membrane and Envelope; s.c., subcutaneous; TCID50, 50% Tissue-Culture Infectious Dose; TM, transmembrane; UTR, untranslated region, VNAb, Virus-Neutralizing Antibody; YF, Yellow Fever Virus.
